# Modulation of Photosynthesis and ROS Scavenging Response by Beneficial Bacteria in *Olea europaea* Plantlets under Salt Stress Conditions

**DOI:** 10.3390/plants11202748

**Published:** 2022-10-17

**Authors:** Estrella Galicia-Campos, Ana García-Villaraco Velasco, Mᵃ Belén Montero-Palmero, F. Javier Gutiérrez-Mañero, Beatriz Ramos-Solano

**Affiliations:** Faculty of Pharmacy, Universidad San Pablo-CEU Universities, 28668 Madrid, Spain

**Keywords:** PGPB, adaptation, salinity, abiotic stress, photosynthesis, hydric stress, oxidative stress, ROS scavenging, antioxidant, induced systemic tolerance (IST)

## Abstract

Climate change consequences for agriculture involve an increase of saline soils which results in lower crop yields due to increased oxidative stress in plants. The present study reports the use of Plant Growth Promoting Bacteria (PGPB) as a tool to modulate plant innate mechanisms of adaptation to water stress (salinity and drought) in one year-old olive plantlets var. Arbosana and Arbequina. Integration of external changes in plants involve changes in Reactive Oxygen Species (ROS) that behave as signals to trigger plant adaptative mechanisms; however, they become toxic in high concentrations. For this reason, plants are endowed with antioxidant systems to keep ROS under control. So, the working hypothesis is that specific beneficial strains will induce a systemic response able to modulate oxidative stress and improve plant adaptation to water stress. Ten strains were assayed, evaluating changes in photosynthesis, pigments, ROS scavenging enzymes and antioxidant molecules, osmolytes and malondialdehyde, as oxidative stress marker. Photosynthesis and photosynthetic pigments were the most affected variables. Despite the specific response of each variety, the favorite targets of PGPBs to improve plant fitness were photosynthetic pigments and the antioxidant pools of glutathione and ascorbate. Our results show the potential of PGPBs to improve plant fitness modulating oxidative stress.

## 1. Introduction

The global increase of temperature and CO_2_ seen in the last decades has dramatically affected rainfall and therefore, water availability for crops. This limitation is not only due to lower water input through rainfall but also to the increase in salt concentration which has turned fertile soils into arid or saline soils, therefore limiting productive soils. In 2050, 50%reduction in productivity is expected due to drought, reaching up to 90% in 2100. Consequently, an increase in food prices, a decrease of world agronomic status and loss of world PIP around 0.3% is foreseen [1]. In view of this, the great challenge for the upcoming decades is to increase agronomic yields under water limiting conditions to feed the increasing world population.

Plants have survived to water stress due to salinity or lack of water along the years thanks to a series of mechanisms that allow a healthy energetic flux. Among these mechanisms are buffering ROS (reactive oxygen species) bursts with ROS-scavenging enzymes and antioxidant molecules, activating signaling cascades for ROS dependent regulatory genes, reversible regulation of proteins with disulfide bridges or phosphoproteins [2,3,4,5,6]. On the other hand, upon stress, ROS are able to activate nuclear gene transcription [7] or trigger systemic signals [8]. Therefore, as keeping ROS levels within a healthy concentration level is key for plant survival, activity of ROS scavenging systems appear as good markers of ROS homeostasis in cells.

Among the innate mechanisms to regulate water homeostasis in plants are (i) to increase internal solute concentration and (ii) to prevent water loss by transpiration. Osmolytes (i.e., proline), epidermal ion carriers in roots as well as ion carriers through xylem, phloem and leaf vacuoles are involved in the first approach, being all of them regulated by activating gene transcription. On the other hand, prevention of water loss affects plant hormonal balance, involving ethylene and abscisic acid levels which results in growth arrest. Either approach involve changes in ROS [9] that need to be brought back to low levels in order to be able to perform as second messengers in future stress events, controlling plant development and adaptation [10,11]. The main mechanisms to control ROS levels are antioxidant systems, both enzymatic and non-enzymatic. The enzymatic system consists of superoxide dismutase (SOD) and of those enzymes in the ascorbate-glutathione cycle (Ascorbate peroxidase (APX), Dehydroascorbate reductase (DHAR), Glutathione reductase (GR)) key to remove ROS and regulate H_2_O_2_ levels [12]. These enzymes are further supported by antioxidant molecules among which are ascorbate, glutathione, phenols and flavonols [13].

Interestingly, a certain overlap in adaptation to biotic and abiotic stress mechanisms has been described, sharing the ROS burst that triggers the systemic signal transduction leading to adaptation [14,15,16]. Therefore, similarly to the improvement of plant protection to biotic stress described for PGPB (Plant Growth Promoting Bacteria) and known as Induced Systemic Resistance (ISR), abiotic stress protection may also be enhanced by PGPB in a process known as Induced Systemic Tolerance (IST). Far from being alternative processes, specific PGPB may trigger simultaneously several plant mechanisms that result in a better adaptation [17]. Among many mechanisms by which PGPB increase IST are water and nutrient exchange, osmolyte accumulation, photosynthesis optimization, regulation of plant hormonal balance and stimulation of antioxidant mechanisms [18,19].

The aim of this study was to evaluate the ability of 10 putative PGPB to improve one-year old *Olea europaea* plantlets adaptation to saline stress, in two varieties, Arbosana (AS) and Arbequina (AQ), after 6 doses of PGPB along 6 months. To achieve our goal, the following parameters were evaluated: (i) changes in photosynthesis as physiological marker; (ii) changes in metabolic markers (photosynthetic pigments, proline, soluble sugars, antioxidant molecules and enzymes); and (iii) malondialdehyde (MDA) as oxidative stress marker, in order to identify the different adaptative mechanisms to salt stress induced by PGPB. 

## 2. Results

This study reports effects of 10 putative PGPB on one year old olive plantlets of Arbosana (AS) and Arbequina (AQ), growing in high saline stress due to soil conditions (6.7 ds m^−1^) and low rainfall, in a 6 month experiment open air in the Guadalquivir Marshes (Spain).

**Figure 1:**
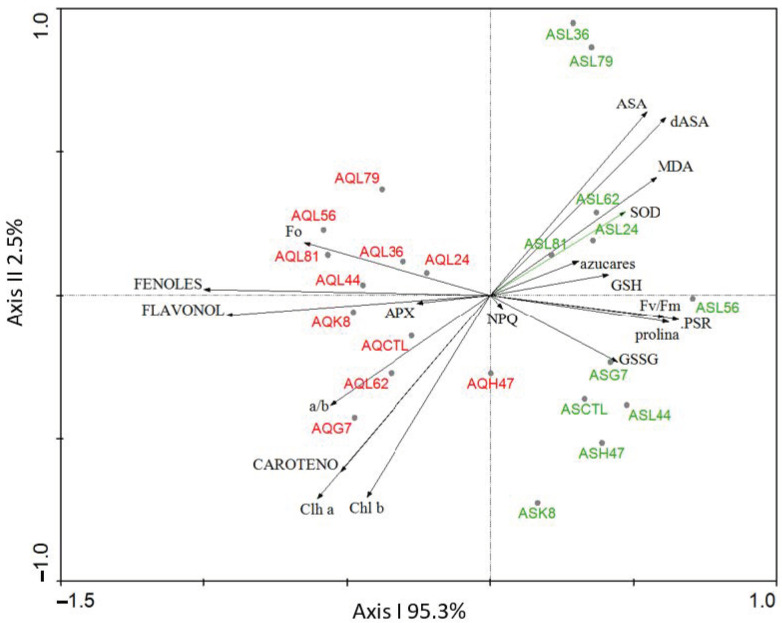
Principal component analysis (PCA) performed with data from physiological, metabolic and oxidative stress markers from AS and AQ inoculated with the 10 PGPB (G7, H47, K8, L24, L36, L44, L56, L62, L79, L81), and non-inoculated controls (CTL). Chl a, Chlorophyll a; Chl b, Chlorophyll b, a/b, ratio Chla/Chlb; CAROTENO, Carotenoids; azucares, Soluble sugars; prolina, proline; FENOLES: Phenols; FLAVONOL: flavonols; SOD, Superoxide Dismutase; APX, Ascorbate Peroxidase; GSH, reduced Glutathione; GSSG, oxidized Glutathione; AsA, reduced Ascorbic; dAsA, oxidized ascorbic acid; F_0_, minimum fluorescence; Fv/Fm, maximal PSII quantum yield; Fv, maximal Fluorescence; NPQ, non-photochemical quenching; PSR: the effective PSII quantum yield. Varianze absorbed by each axis is represented on the figure.

Since the recorded data was abundant, a multivariate analysis was performed initially in order to identify the most relevant factors in our experiment (variety, bacterial strain, marker). Data from all parametres measured on the two varieties treated with the 10 PGPB and controls were analyzed with a principal component analysis (PCA) and results are presented in Figure 1, where axis I absorbs 95.3% of the varianze while axis II absorbs 2.5%. Ordination of samples in the PCA revealed that the genetic variety was the most important factor, as AS samples group towards the positive values of axis I, separating from AQ samples which group towards the negative values of axis I. The factors that determine separation towards the positive values were osmolites (proline and soluble sugars), two photosynthetic parametres (the effective PSII quantum yield, PSR, and the maximal PSII quantum yield, Fv/Fm) and the gluthation pool, while non-enzymatic antioxidants (phenols and flavonols) were responsible for ordination towards the negative values of axis I. Separation along axis II was determined by ascorbic acid pull towards the positive values and photosynthetic pigments (chlorophyll a, b and carotenes) to the opposite end.

In view of the differences of the two varieties, a comparation between them was carried out (Table 1). In short, AS had a higher photosynthetic capacity, with higher chlorophyll b content, higher SOD activity, higher antioxidant molecules content (ascorbate-glutathione cycle) and more osmolytes (proline and soluble sugars) associated to a higher oxidative stress based on MDA (malondialdehyde) values; interestingly, phenolics were in lower concentrations.

Photosynthetic parameters of both varieties inoculated with the 10 strains and the non-inoculated controls appear in Table 2. In AS, only F_0_ and NPQ are affected by inoculation; only 4 strains increase F_0_ (L79, L62, G7, K8) and one (L44) decreases it, while NPQ was increased by all strains. In AQ, all parameters are affected: F_0_ decreases under the influence of 5 strains (L56, L24, L44, K8, H47), up to 10%, while all strains increased Fv/Fm and decreased photosynthetic quantum yield (PSR); finally, only G7 increased NPQ while all other strains decreased it.

**Figure 2:**
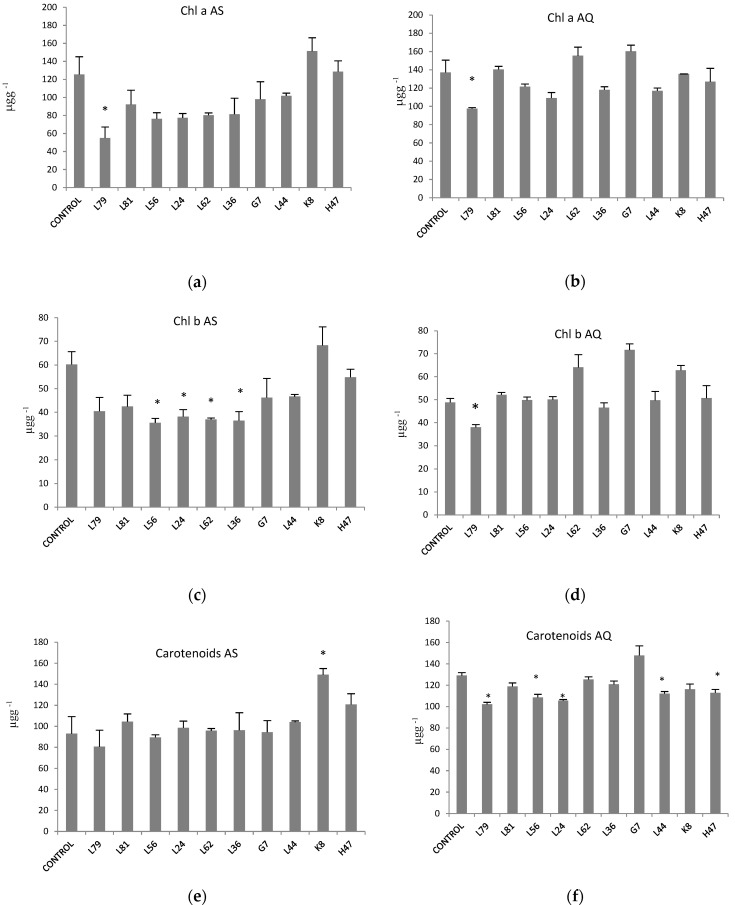
Photosynthetic pigments (μg g^−1^ FW) (**a**) chlorophyll a AS, (**b**) chlorophyll a AQ (**c**) chlorophyll b AS, (**d**) chlorophyll b AQ, (**e**) carotenoids AS and (**f**) carotenoids AQ in leaves of plants inoculated with the 10 strains and non-inoculated controls. Values are the average ± SE (n = 3). Asterisks (*) indicate significant differences with the controls according to T student (*p* < 0.05).

While chlorophyll a and b concentration was similar in both varieties, carotene concentration was higher in AQ (Table 1). In AS, (Figure 2a) only K8 increases chlorophyll a level, while all other strains decrease it, being significant only with L79; in AQ (Figure 2b) values increase with L62 and G7, and decrease with L79 and L24, being significant with L79 only. As regards to chlorophyll b (Figure 2c), strains L56, L24, L62 and L36 significantly decrease values; in AQ (Figure 2d), L79 significantly decreases it, while L62, G7 and K8 increase chlorophyll b content. Finally, carotenoids significantly increased (Figure 2e) with K8 in AS while in AQ (Figure 2f) 5 the trend was to decrease them except for G7 which increased.

**Figure 3:**
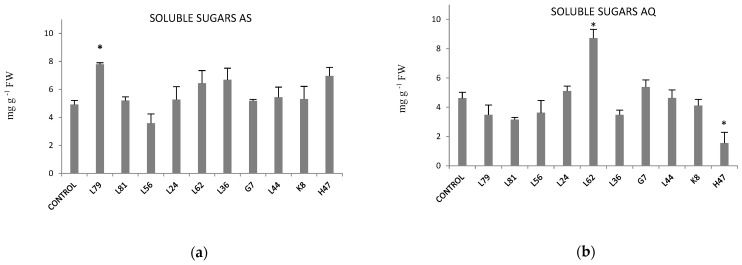
Soluble sugars (mg g^−1^ FW) in (**a**) var. AS and (**b**) var. AQ olive leaves. Values are the average ± SE (n = 3). Asterisks (*) indicate significant differences with the controls according to T student (*p* < 0.05).

Effects of PGPB on osmolytes (proline and soluble sugars) was evaluated. Proline was not affected by any treatment in any of the two varieties (Figure S1) but soluble sugars did (Figure 3). In AS (Figure 3a), L79, L62 and L36 increased, values being significant only with L79, while L56 decreased them. In AQ, (Figure 3b) L62 significantly increased and H47 significantly decreased them. 

**Figure 4:**
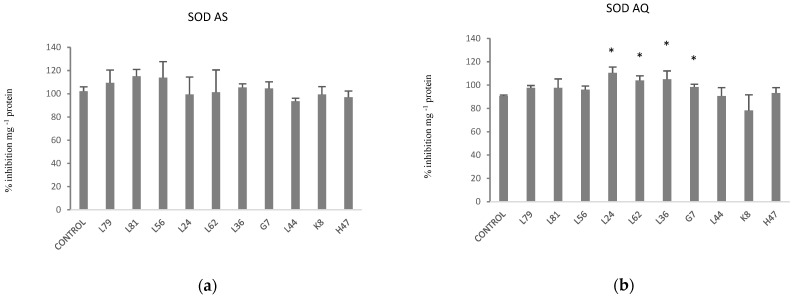
Superoxide dismutase activity (% inhibition mg^−1^ protein) in olive leaves (**a**) var. AS and (**b**) var. AQ, inoculated with the 10 PBPB and non-inoculated controls. Values are the average ± SE (n = 3). Asterisks (*) indicate significant differences with the controls according to *t* student (*p* < 0.05).

As far as modification of antioxidant enzymes activity by bacterial strains, in AS no variation was detected in SOD (Figure 4), while in AQ it was significantly increased by L24, L62, L36 and G7.

**Figure 5:**
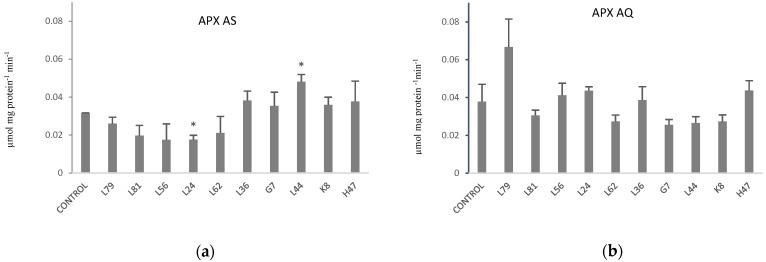
Ascorbate peroxidase activity (µmol mg protein^−1^ min^−1^) in (**a**) AS and (**b**) AQ leaves in plants inoculated with the 10 PGPB and non-inoculated controls. Values are the average ± SE (n = 3). Asterisks (*) indicate significant differences with the controls according to *t* student (*p* < 0.05).

As regards to Ascorbate peroxidase activity (APX), in AS (Figure 5a) a significant decrease with L24 and a significant increase with L44 were registered. In AQ, a non-significant increase with L79 was registered.

**Figure 6:**
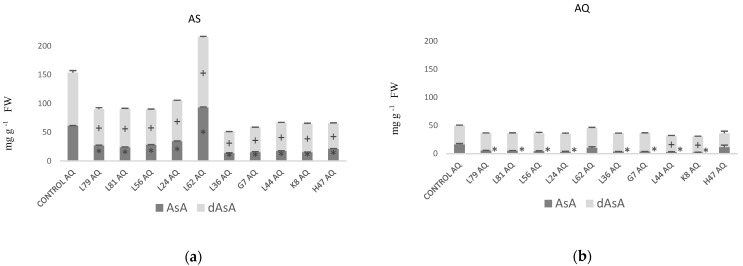
Ascorbate concentration (mg g^−1^ FW) in the oxidized (dAsA) and reduced (AsA) forms in leaves of (**a**) AS and (**b**) AQ, in plants inoculated with the 10 PGPB and the non-inoculated controls. Values are the average ± SE (n = 3). Asterisks (*) or (+) indicate significant differences with the controls according to *t* student (*p* < 0.05) for AsA, and dAsA, respectively.

**Figure 7:**
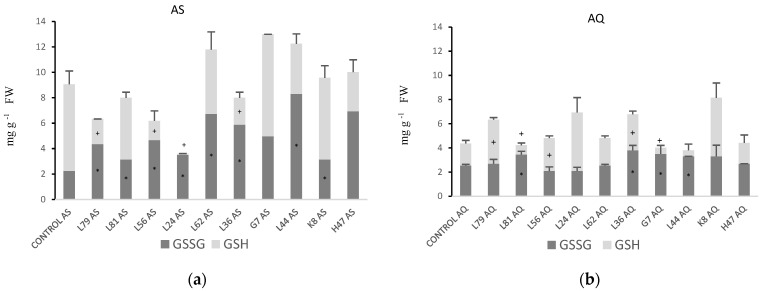
Glutathione concentration (mg g^−1^ FW) of oxidized glutathione (GSSG) and reduced glutathione (GSH) (**a**) in AS and (**b**) AQ, in leaves of plants inoculated with the 10 PGPB and non-inoculated controls. Values are the average ± SE (n = 3). Asterisks (*) and (+) indicate significant differences with the controls according to *t* student (*p* < 0.05) for GSSG and GSH, respectively.

**Figure 8:**
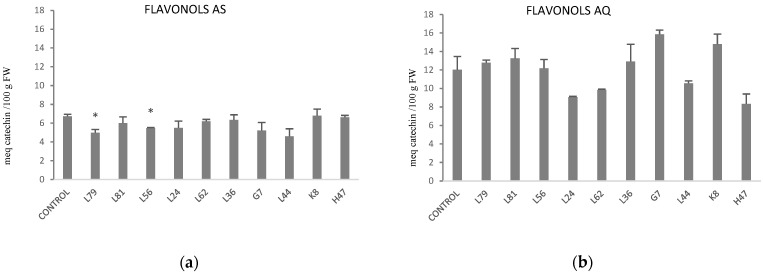
Flavonol concentration (meq catechin/100 g fresh weight) in (**a**) AS and (**b**) AQ leaves of plants inoculated with the 10 PGPB and non-inoculated controls. Values are the average ± SE (n = 3). Asterisks (*) indicate significant differences with the controls according to *t* student (*p* < 0.05).

Antioxidants concentration was affected by inoculation of PGPBs in both varieties (Figure 6, Figure 7 and Figure 8), both in total amounts and in the oxidized/reduced ratio for AsA and glutathione. For the ascorbate pool (AsA/dAsA) is 30% lower in AQ tan in AS (Table 1). The trend in AS was to significantly reduce the total amount of ascorbate, reflected in both species except for L62, that caused a significant increase in both (Figure 6a). In AQ (Figure 6b), all strains except L62, induced a significant decrease in AsA, except K8 and L44 that also decreased dAsA.

Glutathione (Figure 7) is less abundant in AQ than AS, mainly due to the lower concentration of reduced glutathione (GSH) in AQ (Table 1). In AS, the general trend was to significantly increase glutathione, mainly in the oxidized pool, being especially effective L62, G7 and L44; conversely, some strains (L79, L56, L24, L36, H47) significantly decreased GSH. In AQ (Figure 7b), the glutathione pool is significantly increased by L79, L24, L36 and K8, due to increases in GSH, except for L36 that increases both GSH and GSSG; however, strains L81 and G7 modified the balance GSH/GSSG, significantly decreasing GSH.

As regards to the antioxidant molecules phenols and flavonols, phenols were not affected by either treatment in AS or AQ (Figure S2) but flavonols did (Figure 8). Flavonols were almost two-fold higher in AQ than AS (Table 1). While in AS only two strains (L79, L56) triggered flavonoid metabolism, significantly lowering values (Figure 8a), in AQ L24, L62, L44 and H47 followed the same trend while G7 and K8 increased flavonol concentration.

**Figure 9:**
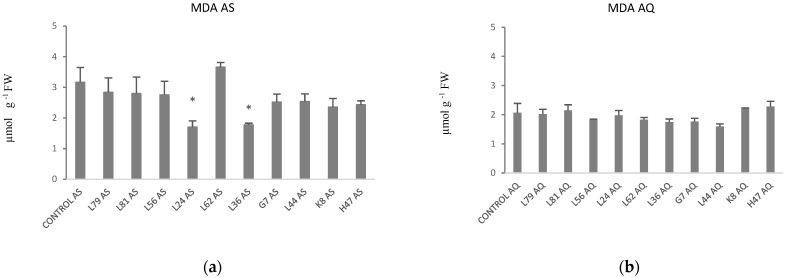
Malondialdehyde concentration (µmol g^−1^ FW), in (**a**) AS and (**b**) AQ leaves of plants inoculated with the 10 PGPB and non-inoculated controls. Values are the average ± SE (n = 3). Asterisks (*) indicate significant differences with the controls according to *t* student (*p* < 0.05).

Finally, concentration of MDA was analyzed being higher in AS than in AQ (Table 1). In AS, all treatments except L62, followed the same trend, tending to lower MDA, although only significant with L24 and L36 (Figure 9a). Interestingly in AQ, this parameter was not affected by any treatment (Figure 9b).

**Figure 10:**
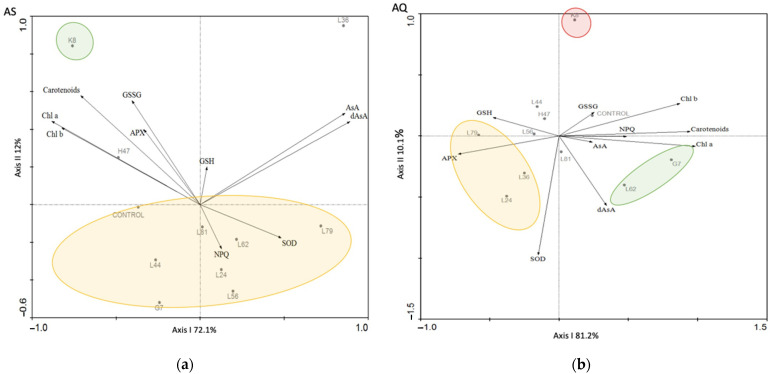
Principal component analysis (PCA) performed with data from physiological, metabolic and oxidative stress markers from (**a**) AS and (**b**) AQ inoculated with the 10 PGPB (G7, H47, K8, L24, L36, L44, L56, L62, L79, L81), and non-inoculated controls (CONTROL). Chl a, Chlorophyll a; Chl b, Chlorophyll b; Carotenoids; SOD, Superoxide Dismutase; APX, Ascorbate Peroxidase; GSH, reduced Glutathione; GSSG, oxidized Glutathione; AsA, reduced Ascorbic; dAsA, oxidized ascorbic acid; NPQ, non- photochemical quenching. Varianze absorbed by each axis is represented on the figure.

Ordination of samples in the PCA revealed that the genetic variety was the most important factor, so individual multivariate analyses were conducted (Figure 10), using only those parameters that were affected by bacterial treatments in both varieties: photosynthetic pigments, NPQ, Superoxide-dismutase, Ascorbate peroxidase, glutathione (oxidized and reduced), asocorbate (AsA/dAsa). In AS (Figure 10a), separation on axis I is driven by pigments towards the negative end and AsA/dAsA towards the positive end; in this figure, axis I accounts for 72.1% of the varianze while axis II accounts for 12%. In AQ (Figure 10b), separation along axis I is driven by pigments towards the positive end and APX and GSH towards the opposite end; in this analysis, axis I accounts for 81.1 % of the varianze and axis II, 10.1%.

## 3. Discussion

The present study shows that photosynthesis and its associated metabolic marker, photosynthetic pigments, are the most affected parameters by PGPB in one-year old olive plantlets. Secondly, ROS regulation mechanisms, both enzymatic and non-enzymatic, are the priority targets, and modification is not necessarily associated to increase their activity or concentration [20]. On the other hand, osmolytes are barely affected suggesting that modification of osmolyte concentration is not a priority mechanism to maintain cell homeostasis at this point of development, despite being priority at later stages [21].

The physiological differences between the two olive varieties were evidenced by the multivariate analysis in which all parameters under study were considered, as samples from each variety grouped together (Figure 1). Photosynthetic performance was different in AS and AQ (Table 1). AS was more efficient under stress conditions as revealed by higher values of maximal potential (Fv/Fm) and real (PSR) photosynthetic efficiency while AQ dissipated more energy (NPQ), confirming that AQ was more sensitive to stress [22,23]. Furthermore, adaptation to oxidative stress relies in different mechanisms in each variety: AS is genetically endowed with stronger SOD activity, a high concentration of ascorbate and glutathione as antioxidant molecules, and high proline content, consistent with its high photosynthetic activity; on the other hand, AQ´s endowment of antioxidant molecules is higher in phenols and flavonols. According to this statement, the potential targets of PGPB to improve adaptation to osmotic stress should be different for each variety [17].

Photosynthetic parameters have been used as indicators of plant responses to biotic stress. More precisely, increases in basal photosynthesis (F_0_) have been reported to indicate an efficient inoculation [24,25], while decreases in F_0_ after inoculation have been interpreted as plant reprogramming for a more relaxed status, that suggests strains protecting the photosynthetic apparatus [26]. Accordingly, fifty percent of strains decreased F_0_ values, while all strains brought up the maximal potential photosynthetic energy (Fv/Fm) to regular healthy values (0.82–0.85), also increasing energy dissipation (NPQ) to release the excess of absorbed energy [22,26,27,28].

Modifications in the photosynthetic process involve photosynthetic pigments. As the role of chlorophylls is to absorb energy, an increase in pigments associated to growth increase upon PGPB treatment could be expected [29]. However, the effect was rather the opposite. Decreases in chlorophyll concentration induced by PGPB has been interpreted as a way to limit energy absorption, resulting in lower oxidative stress [30], a very frequent effect as reported here. As regards to carotenoids (Figure 2), they play several roles either as direct antioxidants [31,32], or directly involved in photosynthesis. In the latter process they play a dual role, either absorbing energy in antennas or dissipating part of the absorbed energy through the xanthophyll cycle [12]; the energy dissipation upon violaxanthin-zeaxanthin conversion is a photoprotective mechanism of PSII, as xanthophylls react with excited chlorophylls preventing ^1^O_2_ formation [33] and releasing energy as heat [9,27]. As antioxidants, carotenoids quench lipid peroxidation products hence stopping oxidative cascade [34,35]. Connecting with the effects of PGPB, the increase in carotenoids could contribute (i) only to energy dissipation, or (ii) to adjust the energy flow increasing absorption in non-stressed conditions while increasing dissipation upon stress, in line to the plant needs to adjust to changing conditions. In summary, the general effect of PGPB in photosynthetic pigments are to decrease chlorophylls while maintaining carotenoid concentration, that is, lowering energy absorption rather than increasing dissipation, in order to slow down the system [30]. Interestingly, only two strains show a different pattern and still, it is true for the two varieties: K8 increases carotenoids supporting the dual role of carotenoids for this case, and L79 that lowers both chlorophylls (56%) and carotenes so, as the energy entrance is decreased, carotenoids do not need to dissipate energy. Finally, an interesting behavior is detected in AQ only, with strains G7 and L62, which increase chlorophyll concentration and therefore, energy absorption but also increase energy dissipation, allowing a higher energy flow through the system with a more relaxed status, preventing ROS formation [35].

Despite all efforts to prevent ROS formation due to their toxicity, non-toxic concentrations are still necessary [12] as they play a role as second messengers in growth and development [36,37], in adaptation to environmental changes [38], activating gene transcription in the nucleus [7], or triggering systemic processes [8]. Based on the different genetic endowment of each variety to keep oxidative stress under control, our data confirms the strong influence on the plant-bacteria interaction [21,26,39], as well as ROS involvement on the response to biotic stress. Each PGPB finds different targets to alter ROS levels, suggesting the existence of different isoenzymes for an optimal adaptation [40]. In AS, PGPBs modify the antioxidant profile targeting APX and ascorbate and glutathione pools as the main antioxidant molecules. Conversely, in AQ PGPBs modify the antioxidant pool targeting SOD, the glutathione and ascorbate pools as well as phenols and flavonols; interestingly, increases in flavonols represent an additional antioxidant mechanism as they reflect UV irradiation [25] and affect lipid peroxidation by increasing phospholipid packaging to prevent ROS diffusion [41]. Increases in SOD, together with decreases in APX and in AsA will result in keeping H_2_O_2_ levels high, as it is the systemic signal to activate IST in the plant, improving adaptation to stress [10]. Beyond the signaling effect, protection of the photosynthetic apparatus to oxidative stress by PGPB is partly achieved by modifying the redox status of AsA and GSH [4]. Modification of antioxidant profiles described above confirm the different strategies of each PGPB to ameliorate oxidative stress due to salinity [42,43], resulting in a better physiological status of the plant due to improved adaptative capacity [44]. 

In summary, PGPB use 3 general strategies to improve plant adaptation to salt stress: (i) Lowering energy absorption by decreasing photosynthetic pigments, which results in a lower oxidative stress and a concomitant decrease of non-enzymatic antioxidants, being this the most frequent option; (ii) Optimizing the energy absorption/dissipation system by increasing chlorophylls and carotenes, without modifying ROS scavenging mechanisms since carotenes are able to play a dual role to absorb and dissipate; unique option for K8 in AQ;. (iii) Optimizing the energy absorption/dissipation system by increasing chlorophylls and carotenes, which results in a higher oxidative stress to be controlled by enhancing antioxidant systems; only two strains in AQ (G7, L62) and K8 in AS. Among all the studied parameters, photosynthetic pigments appear as the most direct marker to detect PGPB effects. Despite the specific response of each variety, the favorite targets of PGPBs to improve plant fitness were the antioxidant pools of glutathione and ascorbate. Our results show the potential of PGPBs to improve plant fitness modulating oxidative stress.

## 4. Materials and Methods

### 4.1. Plant Material 

Two olive varieties were selected Arbosana (AS) and Arbequina (AQ). One-year old plants were bought from a local provider Planta Continental (Rivero de Posada, Córdoba). Plants were transplanted to 5L pots filled with soil from Guadalquivir marshes and peat (3:1).

### 4.2. Bacterial Strains and Inoculum Preparation

The 10 beneficial strains (L79, L81, L56, L24, L62, L36, G7, L44, K8, and H47) assayed in this study were isolated from the rhizosphere of *Pinus pinea* and *P. pinaster* [45]. They were able to produce siderophores (L79, L81, G7, H47), auxins (L56, L24, L44), auxins and siderophores (L62, L36) or auxins and degrade 1-amincyclopropane-1-carboxylate (ACC) (K8). Except for L62, a Gram-positive non-sporulated rod, all other strains are Gram-positive sporulated bacilli [21].

Bacterial strains are kept at −80 °C in nutrient broth amended with 20% glycerol. To prepare inocula, strains were plated (PCA) and incubated for 24 h at 28 °C. Then, they were inoculated on liquid broth (Nutrient Broth (NB) for L62 and Luria Broth (LB) for all other) and incubated 24 h at 28 °C, under shaking. Cultures were diluted to 1 × 10^8^ cfu (Colony forming units) mL^−1^ for inoculation.

### 4.3. Experimental Set Up

Seventy plants from each variety were transplanted to pots and placed on open air at the Guadalquivir marshes (37°11′25.9″ N 6°13′59.3″ W). Ten treatments plus a control were defined for each variety, with 6 plants per treatment. A total of eight inoculations were delivered in October, November, March and April, twice a month, by soil drench, with 400 mL of a bacterial solution (1 × 10^8^ cfu mL^−1^) per plant. Soil moisture was maintained with saline water (electric conductivity 8′2 ds m^−1^), reaching a soil conductivity of 6′07 ds m^−1^. In april 2018, photosynthesis was measured, and leaves were sampled and brought to the laboratory at 4 °C. Leaves from two plants were pulled and constituted an analytical replicate; leaves were powdered with liquid nitrogen to carry on the following determinations: the antioxidant enzymes superoxide dismutase (SOD) and ascorbate peroxidase (APX); the antioxidant molecules phenols, flavonols, Glutathione (oxidized and reduced) and ascorbate (oxidized and reduced); the osmoprotectants proline and soluble sugars; the photosynthetic pigments Chlorophyll a, Chlorophyll b and carotenoids; and malondialdehyde concentration, as oxidative stress marker.

### 4.4. Photosynthesis (Chlorophyll Fluorescence)

Photosynthetic efficiency was determined through the chlorophyll fluorescence emitted by photosystem II. A pulse amplitude modulated (PAM) fluorometer (Hansatech FM2, Hansatech, Inc., UK) was used to measure chlorophyll fluorescence. After dark-adaptation of leaves, a weak modulated irradiation (1 μmol m^−2^ s^−1^) was applied to measure the minimal fluorescence (F_0_; dark-adapted minimum fluorescence). Maximum fluorescence (Fm) was determined from the dark-adapted state delivering a 700 ms saturating flash (9000 μmol m^−2^ s^−1^). The variable fluorescence (Fv) was calculated as the difference between the maximum fluorescence (Fm) and the minimum fluorescence (F_0_). The maximum photosynthetic efficiency of photosystem II (maximal PSII quantum yield) was calculated as Fv/Fm. Immediately, the leaf was continuously irradiated with red-blue actinic beams (80 μmol m^−2^ s^−1^) and after equilibrating for 15 s, Fs was recorded (steady-state fluorescence signal). Then, another saturation flash (9000 μmol m^−2^ s^−1^) was applied to determine Fm’ (maximum fluorescence under light-adapted conditions). Other fluorescent parameters were calculated as follows: the effective PSII quantum yield PSR = (Fm’ − Fs)/Fm’ [46,47]; and the non-photochemical quenching coefficient NPQ = (Fm − Fm’)/Fm’. All measurements were carried out in the 6 plants of each treatment.

### 4.5. Photosynthetic Pigments: Chlorophyll a, Chlorophyll b and Carotenoids 

Extraction was done according to [48], keeping tubes in dark throughout the process. One hundred mg of leaves powdered in liquid nitrogen was dissolved in 1 mL of acetone 80% (*v*/*v*), incubated overnight at 4 °C and then centrifuged 5 min at 10,000× *g* rpm in a Hermle Z233 M-2 centrifuge. One mL of acetone 80% was added to the supernantant and vortexed. Immediately, absorbance at 647, 663, and 470 nm was measured on a Biomate 5 spectrophotometer to calculate chlorophyll a, chlorophyll b, and carotenoids (xanthophylls + carotenes) using the formulas indicated below [47,48]

Chl a (μg g FW^−1^) = [ (12.25 × Abs _663_) − (2.55 × Abs_647_) ] × V(mL)/(g).

Chl b (μg g FW^−1^) = [ (20.31 × Abs _647_) − (4.91 × Abs_663_) ] × V(mL)/(g).

Carotenoids (μg g FW^−1^) = [ ((1000 × Abs _470_) − (1.82 × Chl a) − (85.02 × Chl b))/198] × V(mL)/(g).

### 4.6. Osmoprotectants: Proline and Soluble Sugars

An ethanolic extract was prepared diluting 0.25 g of powder in 5 mL of 70% ethanol (*v*/*v*) incubated at 100 °C for 20 min. The extract was kept at 4 °C until analysis of proline and soluble sugars.

For proline determination 1 mL of ninhydrin reagent freshly prepared (1 g of ninhydrin dissolved in 60 mL of glacial acetic acid, 20 mL of ethanol and 20 mL of water) was mixed with 0.5 mL of the plant ethanol extract and heated at 95 °C for 20 min. Finally, absorbance at 520 nm was measured. Results are expressed as μmol g^−1^ [49].

Soluble sugars were determined according to Yemm and Willis [50]. Briefly, the following reaction was prepared: 3 mL of the reactive (200 mg of antrone + 100 mL of 72% sulfuric acid) and 0.1 mL of the plant ethanol extract. The reaction was incubated in a bath at 100 °C for 10 min. Once it was cold, absorbance was measured at 620 nm. To calculate soluble sugar concentration the following equation was used.

μg g^−1^ = [(Abs620 − 0.016)/0.02]/(g)/1000

### 4.7. Enzymatic Antioxidants: Superoxide Dismutase (SOD) and Ascorbate Peroxidase (APX)

Prior to assessment of enzymatic activities, soluble proteins were extracted. One hundred mg of powder were suspended in 1 mL of 0.1 M potassium phosphate buffer, pH 7.0, containing 2 mM phenylmethylsulfonyl fluoride (PMSF). After sonication for 10 min followed by centrifugation for 10 min at 14,000× *g* rpm, the supernatant was aliquoted, frozen in liquid nitrogen and stored at −80 °C for further analysis of APX, SOD, and proteins. All the above operations were carried out at 0–4 °C. 

To determine the amount of total protein in plant extracts, 250 μL of Bradford reagent, 5 μL samples and BSA (Bovine Serum Albumin) dilutions were inoculated in ELISA 96 well plates and incubated for 30 min at room temperature and then measured using a plate reader at 595 nm. Commercial BSA was used for a calibration curve. Total protein was expressed as mg μL^−1^.

APX was measured as described in [51]. The reaction mixture consisted of 50 mM potassium phosphate buffer, pH 7.0, 0.25 mM sodium ascorbate, 5 mM H2O2 and 100 μL of enzyme extract in a final volume of 1.2 mL. H2O2 was used to start the reaction and ascorbate oxidation was determined by the decrease in A290. The extinction coefficient of 2.8 mM−1 cm−1 was used to calculate activity. One unit of APX activity is defined as the amount of enzyme that oxidizes 1 mmol min−1 of ascorbate under the above assay conditions.

SOD activity was determined as described in the detection kit (SOD Assay Kit-WST, Sigma-Aldrich, Darmstadt, Germany). With this method, xanthine is converted to superoxide radical ions, uric acid, and hydrogen peroxide by xanthine oxidase (XO). Superoxide reacts with WST1 to generate a product that absorbs at around 440 nm. SOD prevents the reduction of WST1 to WST-1formazan, thus reducing the absorption at 440 nm, which is proportional to SOD activity; the rate of the reduction of WST1 with O2 is linearly related to the xanthine oxidase (XO) activity. The unit used for this activity was: % inhibition of WST reduction per mg protein.

### 4.8. Non-Enzymatic Antioxidants: Ascorbate, Glutathione, Phenols and Flavonols

Ascorbate and glutathione were determined according to [52]. An extract was prepared by suspending 1 g leaf powder in 10 mL of 5% metaphosphoric acid and centrifuging for 15 min at 22,000× *g* at 4 °C.

Total ascorbate was determined by fully reducing dAsA to AsA with dithiothreitol (DTT), and then, dAsA was estimated calculating the difference between total ascorbate and AsA. The mixture to determine total ascorbate is as follows: 300 µL supernatant, 750 µL of 150 mM phosphate buffer (pH 7.4) with 5 mM EDTA and 150 µL DTT. After 10 min at room temperature, 150 µL of 0.5% N-ethylmaleimide were added to remove remaining DTT. To determine AsA, a similar mixture was used but DTT and N-ethylmaleimide were replaced by water (300 µL). The reaction was started by adding 600 µL of 10% TCA, 600 µL of 44%ortophosphoric acid, 600 µL of 4% α, α’-dipyridyl in 70% ethanol and 0.3% FeCl_3_ (*w*/*v*). The mixture was vortexed and incubated for 40 min before measuring absorbance at 525 nm. A calibration curve between 0–100 µg mL^−1^ AsA was done.

Total, oxidized (GSSG) and reduced glutathione (GSH) were determined in the supernatant. First, one mL of the supernatant was neutralized with 1.5 mL of 0.5M phosphate buffer (pH 7’5) and 50 µL water to determine total glutathione. Similarly, another mL of the supernatant was also neutralized and supplemented with 50 µL 2-vinylpyridine to mask GSH, by gently shaking to form an emulsion; then, a 60-minute incubation followed and GSSG was determined. GHS concentration was estimated from the difference between total and oxidized glutathione. Glutathione concentration was measured in a 3 mL final volumen reaction containing 0.2 mM NADPH, 100 mM (pH 7.5) phosphate buffer, 5 nM EDTA, DNTB 0.6 mM and 3 units of the enzyme Glutathione Reductase; changes in absorbance at 412 nm for 1 min were recorded. Concentration was calculated from a calibration curve from 0–50 µg mL^−1^.

To determine phenols and flavonols, methanolic extracts were prepared from 0.25 g of leaves (powdered in liquid nitrogen) in 2.25 mL methanol 80%, sonicated for 10 min and centrifuged for 5 min at 5000 rpm.

Total phenols were quantitatively determined with Folin-Ciocalteu agent (Sigma. Aldrich, St. Louis, MO, USA) by a colorimetric method described by Xu and Chang [53], with some modifications; gallic acid was used as standard (Sigma-Aldrich, St. Louis, MO, USA). Twenty µL of extract were mixed with 250 µL of Folin-Ciocalteu 2 N and 750 µL of Na_2_CO_3_ 20% solution. After 30 min at room temperature, absorbance was measured at 760 nm. A gallic acid calibration curve was made (r = 0.99). Results are expressed in mg of gallic acid equivalents per 100 g of fresh weight (FW).

Quantification of total flavonols was done as in [54], using catechin as standard (Sigma-Aldrich, St Louis, MO, USA). One milliliter of the extract was added to a flask of 10 mL with 4 mL of distilled water. Then, 300 µL of NaNO_2_ 5%, and the same volume of AlCl_3_ 10% were added after 5 min. One minute later, 2 mL of NaOH 1 M were added, and adjusted to a total volume of 10 mL with distilled water. The solution was mixed and measured at 510 nm. A catechin calibration curve was made (r = 0.99). Results are expressed as mg of catechin equivalents per 100 g of fresh weigh (FW).

### 4.9. Malondialdehyde

MDA content was determined as in [55]. One hundred mg of leaf powder as suspended in 2 mL trichloroacetic acid 10%. After 2–3 min vortex, it was centrifuged at 20,000× *g* for 30 min at 4 °C. One mL of the supernatant was added to 4 mL 0.5% (*v*/*v*) thiobarbituric acid (TBA) and 20% (*v*/*v*) trichloroacetic acid (TCA). The mixture was heated at 95 °C for 30 min, stopping the reaction on ice. After 10 min centrifugation, absorbance was determined 532 and 600 nm. The MDA content was calculated using the formula: MDA (nmol g FW^−1^) = [(OD532-OD600)]/(ε × W), where FW is the fresh weight and ε the extinction coefficient (155 mM^−1^cm^−1^). Data were expressed as μmol g FW^−1^ (fresh weight).

### 4.10. Statistics

A Principal Components Analysis (PCA) with all the parameters measured for the ten strains was performed with CanocoTM for Windows v.4.5 software (Microcomputer power, Ithaca, NY, USA). To evaluate treatment effects, *t*-Student test were carried out for each variable (Statpgraphcis Centurion XVIII).

## 5. Conclusions

In view of the results presented here, it is evidenced that plant genotype is the most relevant factor that determine plant’s response to stress. As regards to PGPB, all affected photosynthetic pigments, modulating energy flow through the system under saline stress. PGPB use 3 general strategies to improve plant adaptation to salt stress: (i) Lowering energy absorption by decreasing photosynthetic pigments, which results in a lower oxidative stress and a concomitant decrease of non-enzymatic antioxidants, (ii) Optimizing the energy absorption/dissipation system by increasing chlorophylls and carotenes, without modifying ROS scavenging mechanisms since carotenes are able to play a dual role to absorb and dissipate. (iii) Optimizing the energy absorption/dissipation system by increasing chlorophylls and carotenes, which results in a higher oxidative stress to be controlled by enhancing antioxidant systems. Our results show the potential of PGPBs to improve plant fitness modulating oxidative stress although further studies need to be carried out to confirm improvement of plant growth and/or protection to other stress conditions. 

## Figures and Tables

**Table 1:** Physiological and metabolic characterization of AS and AQ and differences between both expressed as % of variation (increase or decrease) of AS compared to AQ. Values are the average ± SE (n = 3). F_0_, minimum fluorescence; Fv, Maximal Fluorescence; Fv/Fm, maximal PSII quantum yield; NPQ, non-photochemical quenching; PSR: the effective PSII quantum yield; Chl a, Chlorophyll a; Chl b, Chlorophyll b; Carotenes; SOD, Superoxide Dismutase; APX, Ascorbate Peroxidase; GSSG, oxidized Glutathione; GSH, reduced Glutathione; AsA, reduced Ascorbic acid; dAsA, oxidized Ascorbic acid; phenols; flavonols; proline; soluble sugars.

Parameters	Control AS	Control AQ	% AS vs. AQ
F_0_	157.67 ± 8.76	216.78 ± 16.84	−27%
Fv/Fm	0.85 ± 0.003	0.77 ± 0.05	10%
PSR	0.77 ± 0.02	0.74 ± 0.02	4%
NPQ	0.10 ± 0.03	0.24 ± 0.02	−59%
Chl a (µg g^−1^)	125.54 ± 19.67	137.02 ± 13.65	−8%
Chl b (µg g^−1^)	60.25 ± 5.39	52.29 ± 5.15	15%
Carotenes (µg g^−1^)	92.93 ± 16.35	134.3 ± 7.91	−19%
SOD (% inhibition g^−1^ protein)	102.2 ± 3.86	90.65 ± 0.9	13%
APX (µmol g^−1^ protein)	0.03 ± 0.00	0.04 ± 0.01	−17%
GSSG (mg g^−1^ FW)	2.24 ± 0.087	2.55 ± 0.087	−12%
GSH (mg g^−1^ FW)	6.82 ± 1.05	1.82 ± 0.26	275%
AsA (mg g^−1^ FW)	61.56 ± 0.15	16.64 ± 1.39	270%
dAsA (mg g^−1^ FW)	92.08 ± 3.82	33.38 ± 0.82	176%
MDA	3.16 ± 0.48	2.06 ± 0.32	53%
Phenols (meq gallic acid per 100 g FW)	739.94 ± 70.42	1031.81 ± 89.63	−28%
Flavonols (meq catechin per 100 g FW)	6.73 ± 0.21	12.02 ± 1.45	−44%
Proline (nmol g^−1^ FW)	0.45 ± 0.02	0.39 ± 0.02	15%
Soluble sugars (mg g^−1^ FW)	4.93 ± 0.60	4.62 ± 0.74	7%

**Table 2:** Photosynthetic parameters of plants from AS and AQ, inoculated with the 10 strains and the non-inoculated controls. Minimum florescence (F_0_). Maximum photosynthetic potential of PSII (Fv/Fm). Photosynthetic quantum yield (PSR) and non-photochemical quenching (NPQ). Values are the average ± SE (n = 3). Asterisks indicate significant differences according to *t*-Student test *p* < 0.05.

	ARBOSANA				ARBEQUINA			
	F_0_	Fv/Fm	PSR	NPQ	F_0_	Fv/Fm	PSR	NPQ
Control	157.67 ± 8.76	0.85 ± 0.003	0.77 ± 0.02	0.10 ± 0.03	216.78 ± 16.84	0.77 ± 0.05	0.74 ± 0.02	0.24 ± 0.02
L79	195.22 ± 17.96	0.83 ± 0.01	0.77 ± 0.03	0.15 ± 0.02	211.33 ± 8.97	0.84 ± 0.01	0.7 ± 0.02	0.23 ± 0.02
L81	150.67 ± 14.71	0.85 ± 0.01	0.8 ± 0.02	0.18 ± 0.03	213.67 ± 8.24	0.82 ± 0.01	0.7 ± 0.02	0.18 ± 0.01
L56	163 ± 0.01	0.85 ± 0.01	0.82 ± 0	0.61 ± 0.01	185 ± 7.62	0.84 ± 0.01	0.75 ± 0.02	0.15 ± 0.01 *
L24	166.67 ± 0.27	0.87 ± 0.01	0.78 ± 0.02	0.12 ± 0.02	190.33 ± 4.48	0.85 ± 0.01	0.72 ± 0.02	0.17 ± 0.01
L62	179.33 ± 5.46	0.85 ± 0.01	0.8 ± 0.01	0.1 ± 0.01	206.67 ± 8.45	0.83 ± 0.01	0.72 ± 0.01	0.23 ± 0.03
L36	170.67 ± 8.02	0.84 ± 0.01	0.74 ± 0.02	0.15 ± 0.02	219 ± 8.26	0.8 ± 0.02	0.67 ± 0.03	0.22 ± 0.03
G7	174 ± 9.81	0.87 ± 0.01	0.78 ± 0.02	0.13 ± 0.01	212.78 ± 13.39	0.82 ± 0.02	0.69 ± 0.02	0.25 ± 0.03
L44	133 ± 18.12	0.85 ± 0.01	0.77 ± 0.02	0.13 ± 0.03	160.67 ± 10.39	0.84 ± 0.02	0.82 ± 0.05	0.2 ± 0.04
K8	179.67 ± 11.47	0.85 ± 0.01	0.77 ± 0.01	0.15 ± 0.02	192.33 ± 9.82	0.82 ± 0.01	0.7 ± 0.04	0.21 ± 0.01
H47	148 ± 21.92	0.86 ± 0.01	0.8 ± 0.01	0.16 ± 0.05	195.75 ± 7.54	0.83 ± 0.01	0.75 ± 0.02	0.16 ± 0.02

## Supplementary Materials

The following are available online at https://www.mdpi.com/article/10.3390/plants11202748/s1, Figure S1: proline contents in As and AQ; Figure S2: total phenols concentration in As and AQ.

## References

[1] Malhi G.S., Kaur M., Kaushik P. (2021). Impact of Climate Change on Agriculture and Its Mitigation Strategies: A Review. Sustainability.

[2] Mittler R., Vanderauwera S., Gollery M., Van Breusegem F. (2004). Reactive oxygen gene network of plants. Trends Plant Sci..

[3] Noctor G., De Paepe R., Foyer C.H. (2007). Mitochondrial redox biology and homeostasis in plants. Trends Plant Sci..

[4] Foyer C.H., Noctor G. (2009). Redox regulation in photosynthetic organisms: Signaling, acclimation, and practical implications. Antioxid. Redox Signal..

[5] Foyer C.H., Bloom A.J., Queval G., Noctor G. (2009). Photorespiratory metabolism: Genes, mutants, energetics, and redox signaling. Annu. Rev. Plant Biol..

[6] Pfannschmidt T., Bräutigam K., Wagner R., Dietzel L., Schröter Y., Steiner S., Nykytenko A. (2009). Potential regulation of gene expression in photosynthetic cells by redox and energy state: Approaches towards better understanding. Ann. Bot..

[7] Woodson J.D., Chory J. (2008). Coordination of gene expression between organellar and nuclear genomes. Nat. Rev. Genet..

[8] Karpinski S., Reynolds H., Karpinska B., Wingsle G., Creissen G., Mullineaux P. (1999). Systemic signaling and acclimation in response to excess excitation energy in Arabidopsis. Science.

[9] Suzuki N., Koussevitzky S., Mittler R., Miller G. (2012). ROS and redox signalling in the response of plants to abiotic stress. Plant Cell Environ..

[10] Garg N., Manchanda G. (2009). ROS generation in plants, boon or bane?. Plant Biosyst..

[11] Huang H., Ullah F., Zhou D., Yi M., Zhao Y. (2019). Mechanisms of ROS regulation of plant development and stress responses. Front. Plant Sci..

[12] Foyer C.H., Shigeoka S. (2011). Understanding oxidative stress and antioxidant functions to enhance photosynthesis. Plant Physiol..

[13] Noctor G., Foyer C.H. (2016). Intracellular redox compartmentation and ROS-related communication in regulation and signaling. Plant Physiol..

[14] Mullineaux P.M., Baker N.R. (2010). Oxidative stress, antagonistic signaling for acclimation or cell death?. Plant Physiol..

[15] Griebel T., Zeier J. (2008). Light regulation and daytime dependency of inducible plant defenses in Arabidopsis: Phytochrome signaling controls systemic acquired resistance rather than local defense. Plant Physiol..

[16] Muühlenbock P., Szechynska-Hebda M., Płaszczyca M., Baudo M., Mateo A., Mullineaux P.M., Parker J.E., Karpińska B., Karpiński S. (2008). Chloroplast signaling and LESION SIMULATING DISEASE1 regulate crosstalk between light acclimation and immunity in Arabidopsis. Plant Cell.

[17] Ilangumaran G., Smith D.L. (2017). Plant growth promoting rhizobacteria in amelioration of salinity stress: A systems biology perspective. Front. Plant Sci..

[18] Kumar A., Singh S., Gaurav A.K., Srivastava S., Verma J.P. (2020). Plant growth-promoting bacteria, biological tools for the mitigation of salinity stress in plants. Front. Microbiol..

[19] Rojas-Tapias D., Moreno-Galván A., Pardo-Díaz S., Obando M., Rivera D., Bonilla R. (2012). Effect of inoculation with plant growth-promoting bacteria (PGPB) on amelioration of saline stress in maize (*Zea mays*). Appl. Soil Ecol..

[20] Gutierrez Albanchez E., García-Villaraco A., Lucas J.A., Gutierrez F.J., Ramos-Solano B. (2018). Priming fingerprint induced by *Bacillus amyloliquefaciens* QV15, a common pattern in *Arabidopsis thaliana* and in field-grown blackberry. J. Plant Interact..

[21] Galicia-Campos E., Ramos-Solano B., Montero-Palmero M., Gutierrez-Mañero F.J., García-Villaraco A. (2020). Management of Plant Physiology with Beneficial Bacteria to Improve Leaf Bioactive Profiles and Plant Adaptation under Saline Stress in *Olea europea* L. Foods.

[22] Yamane K., KawAsAki M., Taniguchi M., Miyake H. (2008). Correlation between chloroplast ultrastructure and chlorophyll fluorescence characteristics in the leaves of rice (*Oryza sativa L*.) grown under salinity. Plant Prod. Sci..

[23] Lutts S., Kinet J., Bouharmont J. (1996). NaCl-induced senescence in leaves of rice (*Oryza sativa* L.) cultivars differing in salinity resistance. Ann. Bot..

[24] Tsai Y., Chen K., Cheng T., Lee C., Lin S., Tung C. (2019). Chlorophyll fluorescence analysis in diverse rice varieties reveals the positive correlation between the seedlings salt tolerance and photosynthetic efficiency. BMC Plant Biol..

[25] Bonilla A., Sarria A., Algar E., Ledesma F.M., Solano B.R., Fernandes J., Mañero F.G. (2014). Microbe associated molecular patterns from rhizosphere bacteria trigger germination and *Papaver somniferum* metabolism under greenhouse conditions. Plant Physiol. Biochem..

[26] Guerfel M., Baccouri O., Boujnah D., Chaïbi W., Zarrouk M. (2009). Impacts of water stress on gas exchange, water relations, chlorophyll content and leaf structure in the two main Tunisian olive (*Olea europaea* L.) cultivars. Sci. Hortic..

[27] Takahashi S., Badger M.R. (2011). Photoprotection in plants, a new light on photosystem II damage. Trends Plant Sci.

[28] Abdallah M.B., Trupiano D., Polzella A., De Zio E., Sassi M., Scaloni A., Zarrouk M., Youssef N.B., Scippa G.S. (2018). Unraveling physiological, biochemical and molecular mechanisms involved in olive (*Olea europaea* L. cv. Chétoui) tolerance to drought and salt stresses. J. Plant Physiol..

[29] Gutiérrez-Albanchez E., Gradillas A., García A., García-Villaraco A., Gutierrez-Mañero F.J., Ramos-Solano B. (2020). Elicitation with Bacillus QV15 reveals a pivotal role of F3H on flavonoid metabolism improving adaptation to biotic stress in blackberry. PLoS ONE.

[30] Trabelsi L., Gargouri K., Hassena A.B., Mbadra C., Ghrab M., Ncube B., Van Staden J., Gargouri R. (2019). Impact of drought and salinity on olive water status and physiological performance in an arid climate. Agric. Water Manag..

[31] Sgherri C.L., Pinzino C., Navari-Izzo F. (1996). Sunflower seedlings subjected to increasing stress by water deficit: Changes in O2—Production related to the composition of thylakoid membranes. Physiol. Plant..

[32] Boo Y.C., Jung J. (1999). Water deficit—Induced oxidative stress and antioxidative defenses in rice plants. J. Plant Physiol..

[33] Srivalli B., Sharma G., Khanna-Chopra R. (2003). Antioxidative defense system in an upland rice cultivar subjected to increasing intensity of water stress followed by recovery. Physiol. Plant..

[34] Burton G.W., Ingold K. (1984). β-Carotene, an unusual type of lipid antioxidant. Science.

[35] Kchaou H., Larbi A., Chaieb M., Sagardoy R., Msallem M., Morales F. (2013). Genotypic differentiation in the stomatal response to salinity and contrasting photosynthetic and photoprotection responses in five olive (*Olea europaea* L.) cultivars. Sci. Hortic..

[36] Foreman J., Demidchik V., Bothwell J.H., Mylona P., Miedema H., Torres M.A., Linstead P., Costa S., Brownlee C., Jones J. (2003). Reactive oxygen species produced by NADPH oxidase regulate plant cell growth. Nature.

[37] Foyer C.H., Noctor G. (2005). Redox homeostasis and antioxidant signaling: A metabolic interface between stress perception and physiological responses. Plant Cell.

[38] Doke N., Miura Y., Sanchez L.M., Kawakita K. (2019). Involvement of superoxide in signal transduction: Responses to attack by pathogens, physical and chemical shocks, and UV irradiation. Causes of Photooxidative Stress and Amelioration of Defense Systems in Plants.

[39] Ramos-Solano B., Algar E., Garcia-Villaraco A., Garcia-Cristobal J., Lucas Garcia J.A., Gutierrez-Mañero F.J. (2010). Biotic elicitation of isoflavone metabolism with plant growth promoting rhizobacteria in early stages of development in Glycine max var. Osumi. J. Agric. Food Chem..

[40] Dietz K.J. (2016). Thiol-Based Peroxidases and Ascorbate Peroxidases, Why Plants Rely on Multiple Peroxidase Systems in the Photosynthesizing Chloroplast?. Mol. Cells.

[41] Arora A., Sairam R., Srivastava G. (2002). Oxidative stress and antioxidative system in plants. Curr. Sci..

[42] Asghari B., Khademian R., Sedaghati B. (2020). Plant growth promoting rhizobacteria (PGPR) confer drought resistance and stimulate biosynthesis of secondary metabolites in pennyroyal (*Mentha pulegium* L.) under water shortage condition. Sci. Hortic..

[43] Khademian R., Asghari B., Sedaghati B., Yaghoubian Y. (2019). Plant beneficial rhizospheric microorganisms (PBRMs) mitigate deleterious effects of salinity in sesame (Sesamum indicum L.), physio-biochemical properties, fatty acids composition and secondary metabolites content. Ind. Crops Prod..

[44] Chin D., Kumar R.S., Suen C., Chien C., Hwang M., Hsu C., Xuhan X., Lai Z.X., Yeh K.-W. (2019). Plant cytosolic ascorbate peroxidase with dual catalytic activity modulates abiotic stress tolerances. iScience.

[45] Barriuso J., Ramos-Solano B., Santamaria C., Daza A., Gutierrez-Mañero F. (2008). Effect of inoculation with putative plant growth-promoting rhizobacteria isolated from *Pinus* spp. on *Pinus pinea* growth, mycorrhization and rhizosphere microbial communities. J. Appl. Microbiol..

[46] Genty B., Briantais J., Baker N.R. (1989). The relationship between the quantum yield of photosynthetic electron transport and quenching of chlorophyll fluorescence. Biochim. Biophys. Acta Gen. Subj..

[47] Lichtenthaler H.K. (1987). Chlorophylls and carotenoids, pigments of photosynthetic biomembranes. Meth. Enzymol..

[48] Porra R., Thompson W., Kriedemann P. (1989). Determination of accurate extinction coefficients and simultaneous equations for assaying chlorophylls a and b extracted with four different solvents: Verification of the concentration of chlorophyll standards by atomic absorption spectroscopy. Biochim. Biophys. Acta Bioenerg..

[49] Carillo P., Gibon Y. (2011). Protocol: Extraction and determination of proline. Prometheuswiki.

[50] Yemm E., Willis A. (1954). The estimation of carbohydrates in plant extracts by anthrone. Biochem. J..

[51] García-Limones C., Hervás A., Navas-Cortés J.A., Jiménez-Díaz R.M., Tena M. (2002). Induction of an antioxidant enzyme system and other oxidative stress markers associated with compatible and incompatible interactions between chickpea (*Cicer arietinum* L.) and *Fusarium oxysporum* f. sp. *ciceris*. Physiol. Mol. Plant Pathol..

[52] Zhang J., Kirkham M. (1996). Antioxidant responses to drought in sunflower and sorghum seedlings. New Phytol..

[53] Xu B.J., Chang S. (2007). A comparative study on phenolic profiles and antioxidant activities of legumes as affected by extraction solvents. J. Food Sci..

[54] Zhishen J., Mengcheng T., Jianming W. (1999). The determination of flavonoid contents in mulberry and their scavenging effects on superoxide radicals. Food Chem..

[55] Lucas J.A., Gutierrez-Albanchez E., Alfaya T., Feo-Brito F., Gutiérrez-Mañero F.J. (2019). Oxidative stress in ryegrass growing under different air pollution levels and its likely effects on pollen allergenicity. Plant Physiol. Biochem..

