# Supplementary material for: Modulation of Photosynthesis and ROS Scavenging Response by Beneficial Bacteria in Olea europaea Plantlets under Salt Stress Conditions
Source: Plants (Basel). 2022 Oct 17;11(20):2748. doi: 10.3390/plants11202748 (PMC9611751; doi:10.3390/plants11202748)
Supplement: Supplementary file 1 [file plants-11-02748-s001.zip › plants-1939269-supplementary.pdf]

Supplementary Materials:

Figure S1: Proline contents in AS and AQ

Figure S2: Total phenols in AS and AQ

Figure S1. Proline concentration.

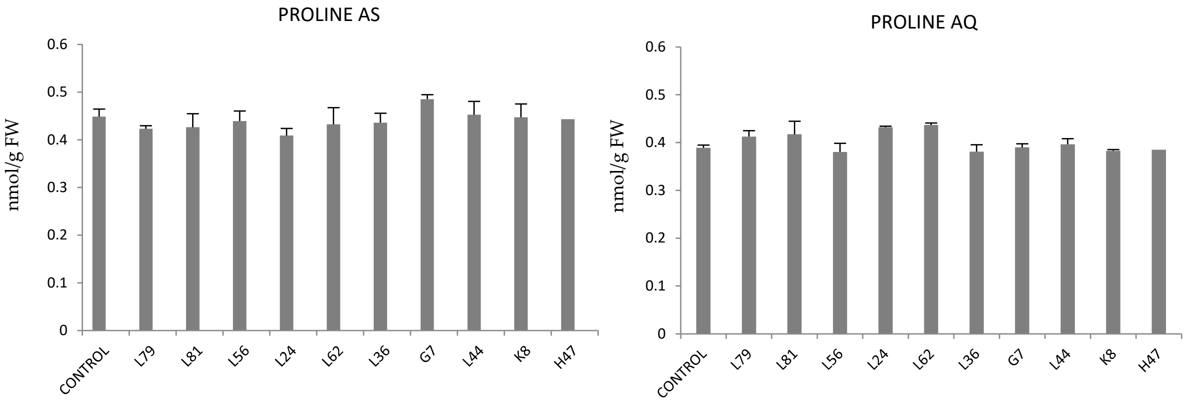

(**a**) (**b**)

Figure S1. Proline (nmol/g fresh weight) in olive leaves **(a)** var. AS and **(b)** var. AQ, inoculated with the 10 PBPB and non-inoculated controls. Values are the average ± SE (n=6). The are no significant differences according to T-STUDENT test.

Figure S2. Total phenol concentration

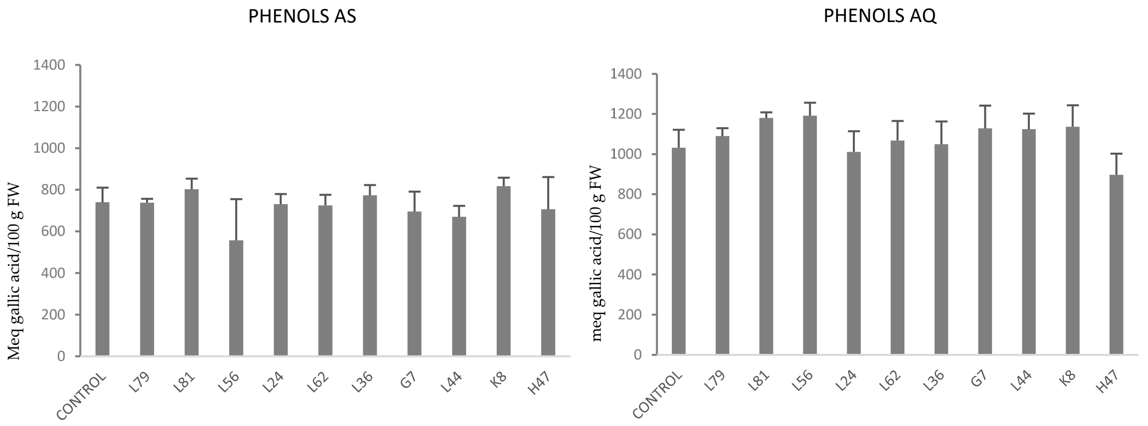

(**a**) (**b**)

Figura S2. Total phenol concentration (meq gallic acid/100 g fresh weight) in olive leaves **(a)** var. AS and **(b)** var. AQ, inoculated with the 10 PBPB and non-inoculated controls. Values are the average ± SE (n=6). The are no significant differences according to T-STUDENT test.
